# Distinct roles of TET-mediated cytosine oxidation in neural stem cell fate decisions

**DOI:** 10.1016/j.stemcr.2025.102614

**Published:** 2025-09-09

**Authors:** Oren Ram

**Affiliations:** 1 Department of Biological Chemistry, Alexander Silberman Institute of Life Sciences, The Hebrew University, Jerusalem, Israel

## Abstract

Ten-eleven translocation (TET) enzymes regulate neural stem cell development via distinct cytosine oxidation steps. This study, by Ebert et al. reveals that hydroxymethylation is crucial for neurogenesis, while formylation and carboxylation drive gliogenesis, offering insights into TET biology and therapeutic potential in neurodevelopmental and neurodegenerative disorders.

## Main text

The development of the mammalian nervous system is orchestrated by epigenetic mechanisms that regulate gene expression programs. One key epigenetic mark is 5-methylcytosine (5mC), deposited primarily at CpG dinucleotides by DNA methyltransferases (DNMT3A/B for *de novo* methylation and DNMT1 for maintenance) (Okano et al., 1999). DNA demethylation, in contrast, is catalyzed by the ten-eleven translocation (TET) family of enzymes, which oxidize 5mC into three derivatives: 5-hydroxymethylcytosine (5hmC), 5-formylcytosine (5fC), and 5-carboxylcytosine (5caC) (Ito et al., 2010). These oxidized forms are removed either actively by thymine DNA glycosylase and base excision repair (Cortellino et al., 2011), or passively when 5hmC prevents the maintenance methylation machinery from acting during DNA replication (Ficz et al., 2011).

These three intermediates are present at low levels, reflecting their dynamic nature; however, 5hmC is comparatively more abundant in the mammalian genome and has therefore been proposed to play additional regulatory roles beyond its function in facilitating demethylation (Wu and Zhang, 2014). Notably, TET enzymes and 5hmC are enriched in the nervous system, specifically at enhancers of neural progenitor cells, pointing to their potential importance in neurodevelopment (MacArthur and Dawlaty, 2021). While single TET gene knockouts in mice result in mild proliferation defects in neural stem cells (NSCs) (Dawlaty et al., 2011), double knockouts (Tet1/2 or Tet1/3) produce more severe phenotypes such as cranial neural tube defects. Complete loss of all three TET enzymes leads to embryonic lethality, making it difficult to study their combined role in later neural development (Ito et al., 2010).

To dissect the individual contributions of oxidized cytosine derivatives, Ebert and a team led by Dawlaty present a comprehensive study distinguishing the biochemical steps of TET enzyme-mediated 5mC oxidation. They utilized a point mutation that permits conversion of 5mC to 5hmC while blocking further oxidation to 5fC and 5caC, introducing it into all three Tet genes to generate Tet1/2/3 triple formylation and carboxylation mutant (Tet-TFoCa) embryonic stem cells (ESCs). These were compared to catalytically dead triple-mutant ESCs (Tet-TMut), which lack all TET enzymatic activity (Dawlaty et al., 2014). The authors show that hydroxymethylation is essential for neuronal competence, while formylation and carboxylation are specifically required for gliogenic competence. Given that TET enzymes and 5hmC levels are often altered in neurodevelopmental and neurodegenerative disorders, these findings provide key insights into TET biology and highlight potential therapeutic targets for neurological disease.

Upon differentiation into NSCs, Tet-TMut cells exhibited a sharp decline in self-renewal, gene expression of core neural markers (Sox1, Pax6, and Otx1), and viability. In contrast, Tet-TFoCa NSCs, while still impaired, retained self-renewal capacity and neural identity for longer durations. These results suggest that hydroxymethylation is necessary for NSC maintenance, while the absence of formylation and carboxylation alone does not fully compromise NSC identity.

Functional differentiation assays revealed further specificity in TET function. Tet-TFoCa NSCs could successfully differentiate into Tuj1^+^ neurons and expressed neuronal markers (Dcx and Tuj1), while Tet-TMut NSCs failed to form neurons and largely died during differentiation. This highlights that 5hmC is required for neuronal competence. However, neither Tet-TFoCa nor Tet-TMut NSCs were able to generate astrocytes or oligodendrocytes, with both lacking expression of key glial markers and failing to induce genes such as Gfap, Mrf, and Sox10. This indicates that formylation and carboxylation are essential for gliogenic competence.

Moreover, RNA sequencing (RNA-seq) supported these functional findings. Tet-TMut NSCs showed far more deregulated genes than Tet-TFoCa NSCs and were transcriptionally distinct. Neuronal differentiation genes, including Neurog1/2, Sox11, and Ascl1, were downregulated uniquely in Tet-TMut NSCs. In contrast, both mutant lines showed common downregulation of glial genes such as Zic3/4/5 and Olig1/2, aligning with the observed glial block.

Epigenomic profiling revealed that regions retaining 5hmC in Tet-TFoCa NSCs were linked to neuronal enhancers and associated with proper expression of genes like Sox11, Npas3, and Zfp521, which were downregulated in Tet-TMut NSCs. These data suggest that 5hmC at enhancers regulates neuronal gene activation. Conversely, promoters and enhancers of glial genes such as Zic3/4/5, Gli2, and Sall2 were hypermethylated and downregulated in both Tet-TMut and Tet-TFoCa NSCs, implicating active demethylation via 5fC/5caC as a necessary step in glial gene expression.

In summary, this study uncovers a dual, non-redundant role for TET enzymatic activities in NSC lineage specification. DNA hydroxymethylation promotes neuronal identity and differentiation, while formylation and carboxylation are critical for glial lineage activation. These findings offer a mechanistic framework for understanding how precise modulation of DNA methylation dynamics influences neurodevelopment and highlight potential targets for therapeutic intervention in neurodevelopmental and neurodegenerative disorders where TET activity is dysregulated (Szulwach et al., 2011).

Future advances in the field will need to address several key technical challenges that are critical for better understanding the role of TET proteins in shaping the epigenomic landscape. These include the inherently dynamic nature of TET enzymes, the low efficiency of 5hmC detection, the underlying heterogeneity of neural differentiation systems, and other known functions of TETs, particularly their link to H3K27me3, an important epigenomic feature in differentiating cells.

Overcoming these challenges will require a combination of innovative approaches, including the integration of single-cell RNA-seq and single-cell assay for transposase-accessible chromatin using sequencing to resolve cellular heterogeneity, mapping H3K27me3 using chromatin immunoprecipitation sequencing (ChIP-seq), and adopting more refined 5hmC mapping strategies. A particularly promising direction is to first enrich for active promoter and enhancer regions using sequential H3K27ac ChIP-seq, as demonstrated by Alajem et al. (2021), and subsequently apply targeted 5hmC profiling across all genetic perturbations presented in this study. Given the specific association of TET enzymes with enhancers and promoters, this sequential approach, H3K27ac ChIP-seq followed by 5hmC mapping, offers a powerful means to enhance both the resolution and contextual understanding of 5hmC’s regulatory roles.

## Declaration of interests

The author declares no competing interests.
